# How will academic meetings look in a future where we combat climate change?

**DOI:** 10.1242/bio.062575

**Published:** 2026-04-17

**Authors:** Teodora Rinciog

**Affiliations:** The Company of Biologists, UK

Discussing science, exchanging ideas, and engaging the new generation of biologists are core values of The Company of Biologists, and we are constantly looking for new ways to do this in an open, inclusive, and environmentally responsible way.

The Company of Biologists' Innovative ideas for the future of sustainable events essay competition gathered many ideas on how innovation will shape the way academics network at scientific meetings in a sustainable future. We received 31 submissions from all around the world (including the UK, France, Malaysia, Thailand, India, Bangladesh) that reflected local and global climate change challenges in organising academic events, and proposed solutions for how to mitigate them. In this article, we will summarise how we expect that innovation will support sustainable practices in the organisation process of academic events.

As seen in Fig. 1, the most cited solutions were virtual reality (VR) and multi-hub systems, which were often cited together. Multi-hub systems are defined as organising the event in multiple decentralised physical hubs around the world, allowing participants to travel to the closest hub and reducing travel emissions. During the event, the hubs will be connected to each other remotely, allowing participants to interact in person with the other participants that are part of their hub, but also remotely with participants from other hubs.

**Fig. 1.**
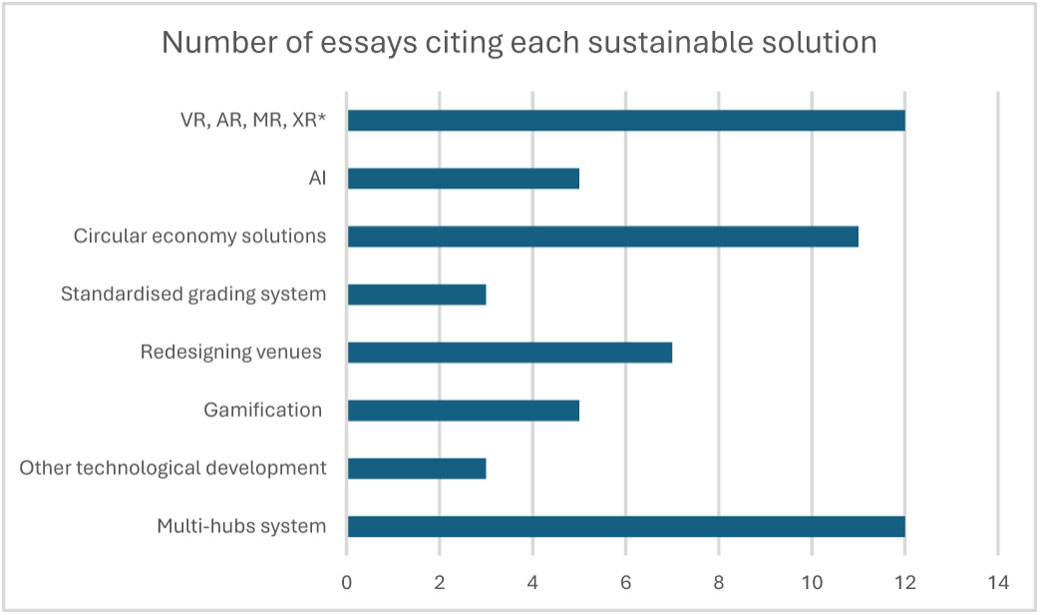
**The main categories of sustainable solutions and the number of essays that have cited them**. *VR=virtual reality; AR=augmented reality; MR=mixed reality; XR=extended reality.

The participants of this competition challenged the classic single-hub system and the biggest environmental issue that it poses: unsustainable international travel. Combining these two elements together, the participants suggested a solution that reduces environmental impact while enhancing social inclusivity, creating solutions for people who live in less accessible regions to join an international event and experience in-person contact through VR solutions. VR brings us a step forward from the classic virtual and hybrid events by enhancing the human experience through solutions that increase engagement and motivation in remote settings (Kirchgessner et al., 2023). While these solutions may currently be more expensive, we highlight that they provide many benefits and discuss the importance of making them more accessible in the future. In a world that is transitioning to using more clean energy, many countries already have the infrastructure to run on renewable electricity sources, minimising the carbon footprint of VR technology's electricity usage. The UK alone has committed to fully transition to clean electricity by 2030 (Department for Energy Security & Net Zero, 2025), while the EU targets a transition to a minimum of 42.5% renewable energy by 2030 (European Commission, n.d.). Compared with that, the development of sustainable fuel for flights progresses more slowly; for example, the EU is expected to raise the usage of sustainable aviation fuel only to 6% by 2030 (European Union, 2023). While an average long haul flight produces around 2 tonne of CO_2_e/person (based on The Company of Biologists’ carbon event calculator), while a video conference creates between 150–1000 g of CO_2_ emissions per hour, depending on the grid powering data centres (World Economic Forum, 2025). While VR becomes increasingly accessible and multi-hub formats more popular, these innovative solutions are particularly important for increasing participation among scientists based in the Global South or remote areas, as research states that in some particular fields of biology, such as genetics, there could still be a large gap in the representation of Global South and Global North scientists, which is particularly obvious when looking at abstract submissions and speaker opportunities. These gaps are mainly attributed to GBP and Nature Index Share, highlighting a persistent global inequality in scientific representation (Zheng et al., 2025). While Zheng's study focused mainly on two large genetics conferences over 25 years, similar gaps might exist in other fields of biology as well. Other highly cited concepts were the circular economy principles. Circular economy is a model of consumption and production that breaks away from the produce-consume-dispose system through three main aspects: extending the life of products, eliminating waste and pollution, and maintaining a regenerative natural system (Ellen MacArthur Foundation, n.d.). Prioritising venues and accommodation that mainly use regenerative energy sources (e.g. solar panels) helps align an event with the circular economy's third principle (i.e. regenerative nature) by indirectly reducing the extraction of non-regenerative resources such as petrol and natural gas for energy (Ellen MacArthur Foundation, 2022). This demonstrates that the academic event industry has the space to flourish in a sustainable economic system (prioritise regenerative resources), where waste is minimised or transformed into resources. Artificial intelligence (AI), while not as highly cited as the previous categories, was described as a tool to support innovation, accelerating new technology development, which will allow scientists to conduct their conferences with a lower environmental impact. Event emissions are mainly driven by the high carbon intensity travel modes that account for an average of 56% of the CO_2_e of an in-person event (Kitamura et al., 2020), and, based on the estimations made with The Company of Biologists' event carbon calculator, they can reach up to 90%. In 2020, it was estimated that a person attending an in-person academic event emits ∼80% of the CO₂e that they should be allowed to emit, based on the global average per person, per year by 2030, in order to limit the rise of global warming to 1.5 °C by 2050 (Jäckle, 2022). Since most recent papers suggest that we will probably exceed the 1.5 °C threshold over the next 20 years (E360 Digest, 2025), this poses an even more urgent need to reduce the carbon footprint per participant attending academic events. Finding the right balance that aligns with social and environmental considerations is challenging, but we are keen to be part of this transition. A significant role in minimising an academic event's environmental impact is reducing the reliance on fossil fuels and increasing renewable energy usage. Reducing these emissions is directly proportional to reducing participants' travelling emissions. This could be achieved by converting in-person events to hybrid/virtual formats and prioritising event locations that are well connected to public transport. For example, in Europe, it would be recommended to choose major rail hub locations such as Frankfurt, over isolated locations such as islands, which would require most of the participants to fly (Jäckle, 2022).

Furthermore, converting to full plant-based menus for an event can reduce the catering carbon footprint by half compared with traditional meat-based menus (Scarborough et al., 2014). However, catering's environmental impact goes beyond its carbon footprint; it also concerns biodiversity loss, soil degradation, freshwater depletion, and deforestation as well (Prencipe et al., 2026; Takacs and Borrion, 2020). Even if, according to The Company of Biologists' carbon event calculator, the catering doesn't have a high carbon footprint compared to other aspects of an event, these other factors mentioned above increase the environmental impact of catering and need to be taken into consideration when building a sustainable menu for an event. For more details, check out The Company of Biologists' guides for how to build a sustainable menu (part 1 and part 2).

Our essay competition received 31 submissions offering interesting and thoughtful solutions to improve the sustainability of academic conferencing. The Company of Biologists' sustainability committee had the challenging task of selecting the top three papers that offered the most innovative solutions for lowering the environmental impact of academic events. Liew Yao Rong (first prize) proposed a complex solution that involves changing participants' behaviour, venue decentralisation, and AI implementation. In the age of emerging AI technologies, implementing AI can aid not only in accelerating new sustainable technology development, but also in gathering data to track the sustainability progress in a more effective and accurate manner.

Liew Yao Rong's solution combines multiple methodologies in one strategy that minimises many of the event's carbon footprint sources covered above by proposing a gamified system on participants' mobiles that encourages responsible behaviour while tracking the carbon footprint of all the participants' activities. It also proposed integrating AI to handle logistics and to find an ideal location for the event based on where the participants were located. This is crucial in international in-person events to calculate the most suitable location, or to identify decentralised multi-hubs for reducing participants' travelling carbon footprint. While AI is an indispensable tool capable of accelerating sustainable development and supporting all its pillars (ecological, social and economic), it also creates the ‘AI green paradox’; while it contributes to sustainable development, using AI comes with its own ecological footprint (Toderas, 2025). AI expansion has increased electricity demand from 1.9% in 2018 to 4.4% in 2023 in the US, with an expected demand of 6.7–12.7% by 2028 (Kim et al., 2026). Thus, AI should be used responsibly to maximise the sustainable development of events, while accounting for and balancing its carbon footprint.

The second prize winner suggested an innovative solution that addressed the other main source of energy consumption of events: venues. Tan Hooi Wen reimagined traditional event venues into venues with integrated bio algae, creating a transition from events that release carbon to events that capture carbon through their activity. While traditional venues are highly energy-consuming, Tan Hooi Wen's proposals reflect how architecture development can correlate with event sustainability by integrating already existing solutions.

Katherine Paine (third prize) highlighted the benefits of a system that brings together data on all upcoming conferences and their estimated carbon footprint, and making it available to the public. As her essay suggests, this information could help academics make more informed decisions about the cost-benefits of participating in an event.

While the competition's winners offered great solutions to reimagine academic meeting organisation, many of the other participants brought insight into what else could be done to be more sustainable. Below, we analysed the most ambitious ideas we received and what their positive impact would be.

## Multi-hub events

While multi-hubs are not a new concept, they are is still not widely adopted as a method to organise events with a lower environmental impact. Yet, when combined with other methods, this could potentially reduce travelling emissions of an event by 78–97% (Jäckle, 2022). As Izebe Evbogame says, “As a teenager growing up in northern Nigeria, my first encounter with science was through a radio show. […] Those moments ignited a dream in me, to one day speak at an international scientific conference. Years later, I found myself boarding a flight to Berlin for a neuroscience symposium. But while the flight connected me with brilliant minds, I couldn't shake the guilt I felt […].”.

The positive impact of multi-hub events extends beyond environmental benefits. As Evbogame explains, “This model democratizes science. According to a UNESCO–UIS survey of global ocean-science conferences held between 2011 and 2018, only 1% of student participants were from sub-Saharan Africa, 2% from Northern Africa and Western Asia, and 2% from Central and Southern Asia.”. The multi-hub event format increases inclusivity and allows participation from less accessible locations, particularly for biologists that have a tight travel budget. As Chukwuemeka Chinaecherem Maryann mentioned, “The shift towards interconnected regional hubs, powered by immersive virtual experiences, isn't just a temporary fix; it's a fundamental reimagining of how scientific exchange can thrive in a travel-conscious era.”.

Sally Lowell reported on a successful test of this idea when co-organising the European Society for Developmental Biology meeting in September 2023 in a multi-hub format in three European cities: Oxford, Paris and Barcelona. Liew Yao Rong also highlighted how this solution was a success at larger-scale events: “the embedded carbon footprint in travelling, for example hotel stays, contributes 20–30% of an attendee's carbon footprint due to the energy required for water, washing, lighting, and cooling (UNWTO, 2021). […] A similar approach was taken at the COP26 summit, where technology was used to build off-site zones using modular pavilions in several locations to include more participants and minimise travel by 30% (UNFCCC, 2021).”.

## VR

VR was one of the most frequently mentioned ideas in the submitted essays as a solution to reduce carbon emissions from event travel while maintaining the benefits of in-person interaction. We acknowledge that hybrid and virtual events could detract from the human experience of in-person events, and this was our fundamental motivation to create the Fund for Innovation in Sustainable Conferencing, which supports innovative solutions that enhance the ‘human’ experience in virtual meetings. While reality-enhancing technologies are not yet widely used in the events industry, they do not only reduce emissions linked to travel, but also “improve communication by reducing the pitfalls of social anxiety experienced by people during in-person meetings such as awkward encounters, moments of self-doubt, or the pressure to speak up without feeling fully prepared, which allows for more effective communication” (Fathimath Yania Shahid). As Kirchgessner et al. (2023) mentioned, VR increases motivation independently compared with traditional virtual conferencing settings (e.g. Zoom-based settings), regardless of whether participants have used VR technology before. The participants reported higher enjoyment and excitement in VR settings, with no difference in stress or focus when engaging compared with traditional remote settings.

## Gamification and participant engagement

Engaging participants in accounting for the emissions of an event will be a great step towards identifying the sources that could be reduced more quickly. Using a gamifying system to involve participants in the data collection process will not only improve accuracy and reduce data collection time but will also encourage behaviour change by raising awareness about global warming and, as Akrish Bhandari mentioned in their essay, by “promoting pro-environmental behaviour by using motivation, social connection, and feedback mechanisms (Landers, 2019)”.

A gamified app where participants can input their activity (travelling mode, meal options, etc.) so that their data are transformed into CO_2_e in real time. As Peter Whitehouse said in his essay, “The application would aim to ensure that no one is excluded from the sustainability discourse. It requires a basic smartphone, is intentionally low-energy, and possesses a significant level of reusability.”.

Akrish Bhandari describes how the app could increase efficiency by using AI and recording historical data for meal preferences. Using AI, this data could be used to adjust the number of meal portions requested for the event and reduce food waste. As Akrish Bhandari describes “replacing a single beef meal with a vegetarian alternative can reduce greenhouse gas emissions by up to 2.5 kg of CO₂ equivalent (Poore & Nemecek, (2018).”.

## Redesigning venues

Architecture innovation will reshape event venues by creating spaces that are more energy efficient and prioritise renewable sources. Tan Hooi Wen (second prize) proposes integrating algae in a system that absorbs energy instead of releasing it: “Zero-Footprint Hub is a microalgae biopanel system that uses photosynthesis to generate electricity. Non-toxic algae like *Chlorella vulgaris* absorb sunlight and produce a steady flow of electrons, powering low-energy devices such as sensors and microprocessors (Cambridge University, 2022). Simultaneously, the algae absorb CO₂, making the system carbon-negative through natural sequestration”. While buildings with such systems already exist (e.g. BIQ House in Germany), this technology is more of a concept than a practical solution to implement today, but it brings us the vision of how event venues could be designed in the future.

Along with smart energy control, venues could implement kinetic energy capture systems through the floors, which would transform walking or standing into clean energy (current systems like Pavegen generate up to 5 watts per tile). As Emmanuel John Andoy Galvan describes these systems, “by covering entrance halls, buffet areas, exhibition booths, lecture halls, and rest spaces with this system, it can maximise energy capture, aligning with SDG 7.”. AI could also be used for automatic energy control that can turn off the lights and heating when detecting that people have left a certain room (i.e. during a break). While the costs for kinetic floors are higher than those of traditional pavements, the technology is already available – a British company, Pavegen, has completed over 300 projects in 37 different countries (Pavegen, n.d.), including the World EXPO in Astana; Broadgate, London; Smart City Development, Bangalore; Abu Dhabi Airport, UAE; Kiin Energy, Mexico; True Digital City, Bangkok; Dupont Circle, USA; H6 Conet, Hong Kong; Chelsea Flower Show, London; University of Birmingham, UK; and Oxford Street, London (Sustainable Avenue, n.d.).

Reusable mobile venues minimise the travel footprint and increase inclusivity. Choong Jing Yang highlights how building a mobile venue based on circular economy principles, using “lightweight, durable materials for easy transportation and quick setup. The hub includes collapsible aluminium honeycomb walls, solar panels for power, and augmented reality (AR) display walls for interaction”, which would improve connection with other hubs.

## Standardised grading systems

There aren't many internationally recognised standards for sustainable practices in the event industry.

Katherine Paine (third prize) proposed a system that would aid academics in ranking conferences based on their carbon footprint, making informed decisions about the environmental trade-off costs of attending a conference. As Katherine Paine mentioned, “Researchers wishing to attend an event could search a database by inputting their location and interests. […] Entries that match their criteria would be ranked based on sustainability and the researcher could browse through, with each event's characteristics and approach to sustainability being highlighted. And so, an imaging workshop accessible to the researcher by train would rank higher than one that requires air travel.”.

These criteria would not only help scientists make more informed decisions but would also centralise data reporting in the academic event industry. As Nur Iman Binti Mohd Azahari suggests, “A Green Accreditation System for scientific conferences […] aims to evaluate and certify events based on their environmental and social performance, offering a practical tool for change. […] In doing so, the scientific community can lead by example, advancing both knowledge and climate action together.”.

However, it would be a concern whether the event's quality or speakers' choices could weigh more in the decision process than the sustainability score. The goal of a scientific meeting is to offer quality content and networking to participants, so it is crucial that the organisers balance the sustainability contribution without compromising the quality of content. While some scientists might not take into consideration sustainability as a decision criterion, making it visible will still raise awareness about the environmental impact of a meeting and start a conversation about it.

## Technology development and circular economy

While some of the solutions we received were highly advanced technologically, others proposed repurposing old technology for sustainability purposes. One example was described by Liew Yen Chyi's essay as a “natural projector, inspired by the camera obscura, which uses natural sunlight to project visuals, reducing reliance on artificial energy sources during scientific events.”. Events also generate large amounts of waste; Nicolaus Putra highlights that “a standard three-day conference hosting 1000 attendees would generate around 5670 kg (12,500 lbs) of waste, equivalent to the weight of four compact cars (attendease24, 2024)”. A first step to approach these issues is by building up solutions using circular economy principles as a foundation. Applying circular economy principles maximising renewable sources of energy, minimising waste, and redesigning the systems in an innovative manner will help create events with reduced environmental impact.

## Conclusions

Communication is key for scientific development. Scientific meetings do not only help maintain good scientific practices, but also foster inclusion within the scientific community, creating opportunities to share knowledge and build new collaborations that accelerate scientific progress. While conferencing brings many benefits to the community of biologists, it also comes with sustainability challenges that we aim to address to ensure that scientific meetings can continue to deliver quality content and networking, but with a minimised environmental impact (Lowell et al., 2022).

This competition revealed a global perspective on how we could redesign the way academic events are conducted to minimise their environmental impact.

The vision of sustainable academic events was designed through maximising emerging technologies, but also by creating more structured systems to track and identify the sources of emissions better.

Academics could revisit the structure of in-person events where everyone travels to one single place to meet and opt for virtual or hybrid alternatives. There will be more investment in integrating reality-enhancing technologies to improve human connections online. AI will also facilitate logistics, data gathering and analysis processes. Engaging the participants and sharing the responsibility for emissions with them will also become a significant step in improving data quality and further reducing emissions.

An international system for estimating the emissions of an event and shared transparently with academics, will help scientists make a more informed decision about the cost-benefit of attending a conference.

Architecture development will also play a crucial role in reducing the carbon footprint of events. In the future, it is envisioned that conferences will adapt more to people's needs and event location choices will not reduce accessibility but enhance it. Building mobile, energy-efficient venues with sustainable materials and renewable energy systems will support academic meetings in becoming more inclusive and less costly for the environment. Finally, switching to fully renewable energy sources will significantly reduce the carbon footprint of the events of the future.

## Resources

The Company of Biologists launched its Sustainability Initiative in 2020 to support the biology community in the transition away from traditional models to more sustainable interaction models. This initiative shares financial support through The Fund for Innovations in Sustainable Conferencing. Free advice and case studies are easily accessible on our website, and the newly launched event carbon calculator can estimate the carbon footprint of an event and offer tailored recommendations for improvement in less than five minutes.

The success of our Essay competition: Innovative ideas for the future of sustainable events tells us that there are plenty of ideas out there to improve conference sustainability. If you have any ideas of your own, please do get in touch with us – we at The Company of Biologists are here to help put your ideas into practice as we move together towards a more sustainable future.

## References

[BIO062575C1] attendease24. (2024, May 30). Save Money and Trees: How to Run Sustainable Events | EventUp Planner. EventUp Planner. https://eventupplanner.com/how-to-run-sustainable-events/?utm_source=chatgpt.com

[BIO062575C2] Cambridge University (2022). Algae-powered computing: Scientists create reliable and renewable biological photovoltaic cells. Available at: https://www.cam.ac.uk/stories/algaepoweredcomputing (Accessed: 26 November 2025).

[BIO062575C3] Department for Energy Security and Net Zero (2025). Clean Power 2030 Action Plan: A new era of clean electricity – main report. 15 April. Available at: https://www.gov.uk/government/publications/clean-power-2030-action-plan/clean-power-2030-action-plan-a-new-era-of-clean-electricity-main-report (Accessed: 10 February 2026).

[BIO062575C4] E360 Digest (2025). ‘World Likely to Breach 1.5-Degree Target, Research Finds’. Available at: https://e360.yale.edu/digest/1.5-goal-threshold-research. (Accessed: 31 March 2026).

[BIO062575C5] Ellen MacArthur Foundation (2022). Circular economy principles: Regenerate nature. Available at: https://www.ellenmacarthurfoundation.org/regenerate-nature (Accessed: 9 March 2026).

[BIO062575C6] Ellen MacArthur Foundation (n.d.). What is the meaning of a circular economy and what are the main principles?. Available at: https://www.ellenmacarthurfoundation.org/topics/circular-economy-introduction/overview (Accessed: 10 February 2026).

[BIO062575C7] European Commission (n.d.). Renewable energy targets. Available at: https://energy.ec.europa.eu/topics/renewable-energy/renewable-energy-directive-targets-and-rules/renewable-energy-targets_en (Accessed: 10 February 2026).

[BIO062575C8] European Union (2023). REGULATION (EU) 2023/2405 of the European Parliament and of the Council, 18 October. Available at: https://eur-lex.europa.eu/legal-content/EN/TXT/?uri=celex%3A32023R2405 (Accessed: 10 February 2026).

[BIO062575C9] Jäckle, S. (2022). The carbon footprint of travelling to international academic conferences and options to minimise it’. In *Academic Flying and the Means of Communication* (ed. K. Bjørkdahl and A. S. Franco Duharte), pp. 1-20. Singapore: Palgrave Macmillan.

[BIO062575C10] Kim, M. K., Yoo, T. A. and Chung, J. B. (2026). Toward sustainable AI: a scoping review of carbon footprint and environmental impacts across training and inference stages. *IEEE Access* 14, 1-20. 10.1109/ACCESS.2026.3659894

[BIO062575C11] Kirchgessner, E., Sothers, M., Aravena, V., Baloian, N. and Zurita, G. (2023). The Mind in Virtual Meetings: Comparing VR and Video Conferencing Environments Through Experiential Impact Assessment and EEG Analysis’, in Bravo, J. and Urzáiz, G. (eds) Proceedings of the 15th International Conference on Ubiquitous Computing & Ambient Intelligence (UCAmI 2023). Cham: Springer, Lecture Notes in Networks and Systems, 835, pp. 1-15. 10.1007/978-3-031-48306-6_26

[BIO062575C12] Kitamura, Y., Karkour, S., Ichisugi, Y. and Itsubo, N. (2020). Carbon footprint evaluation of the business event sector in Japan. *Sustainability* 12, 5001. 10.3390/su12125001

[BIO062575C13] Lowell, S., Downie, A., Shiels, H. and Storey, K. (2022). The future of conferences. *Development* 149, dev200438. 10.1242/dev.200438 34982149 PMC7618516

[BIO062575C14] Pavegen (n.d.). Every step generates a powerful connection. Available at: https://www.pavegen.com/ (Accessed: 3 February 2026).

[BIO062575C15] Prencipe, S. A., Vinci, G., Ruggeri, M., Savastano, M., Billi, A. and Maddaloni, L. (2026). Sustainable diets in collective catering: developing a menu scoring system to evaluate environmental performance. *Sustainability* 18, 1660. 10.3390/su18031660

[BIO062575C16] Scarborough, P., Appleby, P. N., Mizdrak, A., Briggs, A. D. M., Travis, R. C., Bradbury, K. E. and Key, T. J. (2014). Dietary greenhouse gas emissions of meat-eaters, fish-eaters, vegetarians and vegans in the UK. *Clim. Change* 125, 179-192. 10.1007/s10584-014-1169-1 25834298 PMC4372775

[BIO062575C17] Sustainable Avenue (n.d.). These floor tiles harvest the kinetic energy from human footsteps. Available at: https://sustainableavenue.com/project/these-floor-tiles-harvest-the-kinetic-energy-from-human-footsteps/ (Accessed: 3 February 2026).

[BIO062575C18] Takacs, B. and Borrion, A. (2020). The use of life cycle-based approaches in the food service sector to improve sustainability: a systematic review. *Sustainability* 12, 3504. 10.3390/su12093504

[BIO062575C19] Toderas, M. (2025). Artificial intelligence for sustainability: a systematic review and critical analysis of AI applications, challenges, and future directions. *Sustainability* 17, 8049. 10.3390/su17178049

[BIO062575C20] UNWTO. (2021). Hotels and climate change: Carbon footprint assessment for the accommodation sector (2nd ed.). https://www.unwto.org/sustainable-development/tourism-emissions-climate-change

[BIO062575C21] UNFCCC (2021). COP26 Sustainability Report. United Nations Framework Convention on Climate Change. Available at: https://unfccc.int/sites/default/files/resource/COP26-Sustainability-Report_Final.pdf (Accessed: 10 February 2026).

[BIO062575C22] UNESCO-IOC (2020). *Global Ocean Science Report 2020: Charting Capacity for Ocean Sustainability*. Paris: UNESCO. Available at: https://unesdoc.unesco.org/ark:/48223/pf0000375147 (Accessed: 10 February 2026).

[BIO062575C23] World Economic Forum (2025). The carbon footprint of remote work: Designing low carbon digital collaboration’, 24 September. Available at: https://www.weforum.org/stories/2025/09/the-carbon-footprint-of-remote-work-designing-low-carbon-digital-collaboration/ (Accessed: 10 February 2026).

[BIO062575C24] Zheng, H., Wang, Y., Tanigawa, Y., Ong, J. S., MacGregor, S., Liang, L., Kellis, M. and Han, X. (2025). Disparities and trends in global representation of human genetics conferences: a 26-year longitudinal study of ASHG and ESHG. *medRxiv*. 10.1101/2025.08.12.25333491

